# Does integrating pelvic tilt change from sitting to standing in functional 3D planning enhance early complications of robotic-assisted total hip arthroplasty?

**DOI:** 10.1302/2633-1462.74.BJO-2025-0381.R1

**Published:** 2026-04-27

**Authors:** Pascal Kouyoumdjian, Anthony Marquant, Thomas Grosso, Remy Coulomb

**Affiliations:** 1 Department of Orthopaedic Surgery, University Hospital of Nîmes, Faculty of Medicine, University of Montpellier, Nîmes Cedex, France; 2 University Mechanical and Civil Engineering Laboratory, Montpellier, France; 3 University of Montpellier-Nîmes, Montpellier, France

**Keywords:** Robot-assisted total hip arthroplasty, Functional planning, Pelvic tilt, Lumbopelvic complex, 3D planning, Collision modelling, Safe zone, Functional outcomes, Implant positioning, Mako robotic assistance, robotic-assisted total hip arthroplasty, reoperations, patient acceptable symptom state (PASS), Anesthesiologists, kinematics, Periprosthetic fractures, BMI, Oxford Hip Score, Forgotten Joint Score

## Abstract

**Aims:**

While robotic-assisted total hip arthroplasty (THA) improves implant positioning accuracy, it often overlooks dynamic lumbopelvic parameters, such as pelvic tilt changes between sitting and standing. These factors are increasingly recognized as critical in preventing early complications, such as impingement and dislocation. The aim was to determine whether integrating pelvic tilt variations into functional 3D planning reduces complications and improves outcomes following THA.

**Methods:**

In this retrospective cohort study, 656 patients underwent robotic-assisted THA using either conventional CT-based planning (V3) or functional kinematic-based planning (V4). The V4 group incorporated lumbopelvic kinematics, specifically pelvic tilt in seated and standing positions, into implant planning via a collision model. Outcomes at ≥ one year included complication rates (dislocations, periprosthetic fractures, reoperations) and functional outcomes (visual analogue scale, Harris Hip Score, Oxford Hip Score, Forgotten Joint Score).

**Results:**

Compared with V3, the V4 group had significantly fewer overall complications (2.8% vs 8.4%, p = 0.007), no dislocations (0% vs 1.7%, p = 0.048), and fewer reoperations (p = 0.017). Reoperation-free survival at two years was higher in the V4 group (97.2% vs 94.1%). Functional outcomes were significantly improved across all scores, with more patients achieving patient acceptable symptom state thresholds (p < 0.05). In multivariate analysis, functional planning (V4) was the only independent protective factor against reoperation, while age, sex, BMI, American Society of Anesthesiologists grade, smoking, approach, and insert type were not significantly associated.

**Conclusion:**

Functional planning that incorporates the kinematics of the lumbopelvic complex (V4) was the only independent protective factor against reoperation, highlighting the importance of integrating lumbopelvic dynamics into robotic-assisted THA.

Cite this article: *Bone Jt Open* 2026;7(4):584–592.

## Introduction

Total hip arthroplasty (THA) is a highly successful procedure, with satisfaction rates around 85%,^1^ although up to 8% of patients remain dissatisfied.^2,3^ Malposition of implants remains a major cause of early complications and revision.^4^ Dual-mobility cups reduce instability,^5^ but do not compensate for the influence of the lumbopelvic complex (LPC), where unrecognized spinal stiffness or abnormal pelvic tilt can increase the risk of impingement and dislocation. Pelvic tilt varies significantly between standing and sitting, modifying functional cup orientation and affecting hip stability.^6^ Balancing femoral offset, length, and version is also influenced by these dynamic parameters. Recent studies emphasize the importance of integrating LPC kinematics into preoperative planning.^7,8^

Moreover, dual-mobility designs do not prevent the risk of impingement when malposition occurs. Unrecognized spinal stiffness can therefore contribute to instability and early implant failure.^9^ Recent studies have therefore underscored the importance of 3D planning that incorporates lumbopelvic kinematics.^7,10^

Robotic-assisted THA improves accuracy in implant positioning, but conventional CT-based planning does not account for pelvic mobility.^11-13^ The integration of lumbopelvic kinematics into planning represents a further step, allowing simulation of impingement and dislocation risks in functional positions.^14-16^ The most recent software updates of the MAKO (Stryker, USA) robotic system incorporate lumbopelvic mobility into planning through a collision model, offering a patient-specific ‘functional’ approach to implant orientation^8,17,18^ influenced by the spino-pelvic segment balance above the pelvis.^8^ This model used in the updated software (V4), which integrates sacral slope in seated and standing positions, evaluates bone-bone, bone-implant, and implant-implant impingement throughout the simulated functional range of motion. This allows the software to identify mechanical conflicts during flexion, extension, and rotational movements.

While patient- and surgery-related factors (age, sex, BMI, American Society of Anesthesiologists (ASA) grade,^19^ smoking, approach, and implant type) have been linked to complications, the specific impact of functional planning has not been evaluated in large comparative series.^6^

Although the expected primary effect of functional planning is on instability, the software update could theoretically influence other complications; for example, through improved workflow efficiency. Therefore, overall complication-free survival was selected as the primary endpoint.

The primary aim of this study was therefore to compare complication-free survival at a minimum of one year between robotic-assisted THA performed with conventional planning (V3) and with functional LPC-based planning (V4). A multivariate analysis was conducted to isolate the specific effect of functional planning on reoperation risk. The secondary aim was to compare functional outcomes between the two groups

## Methods

This single-centre retrospective study analyzed a prospective cohort of patients undergoing robotic-assisted total hip arthroplasty (MAKO). Outcomes were compared between conventional 3D planning (V3) and functional planning (V4), which integrates pelvic tilt in sitting, standing, and supine positions. Data were prospectively collected between December 2018 and December 2022 and retrospectively analyzed. The primary endpoint was to assess the impact of lumbopelvic kinematic integration (V4) on hip pain, function, and short- to mid-term complications. The study was approved by the institutional review board (IRB no. 17.011), and all patients provided written informed consent.

### Study population and demographic data

All consecutive patients aged over 18 years who underwent primary THA with at least one year of follow-up were included. Demographic data (sex, age, height, weight, BMI, ASA grade) and lifestyle factors (tobacco, alcohol, preoperative opioid or other substance use) were recorded. Patients operated on for traumatic indications were excluded.

A total of 406 patients in the V3 group were compared with 250 in the V4 group. Demographic characteristics were broadly comparable between groups in terms of age, sex, BMI, ASA grade, and previous hip or spine surgery (Table I).

**Table I.** Demographic and surgical characteristics of patients undergoing robotic-assisted total hip arthroplasty with V3 and V4 planning software.

Variable	V3 group (n = 406)	V4 group (n = 250)
Male sex, n (%)	192 (47.3)	124 (49.7)
Mean age, yrs (SD; range)	67.9 (11.86; 26.1 to 93.5)	68.6 (10.22; 41.7 to 91.7)
**ASA grade, n (%)**		
I	92 (22.7)	53 (21,2)
II	276 (68.0)	140 (56.0)
III	35 (8.5)	50 (20.0)
IV	3 (0.7)	7 (2.8)
**Tobacco use, %**		
Active	16.3	16.0
None	61.0	64.7
Weaned	22.7	19.3
Mean BMI, kg/m^2^ (SD; range)	27.0 (5.0; 15.6 to 43.3)	27.4 (4.9; 18.0 to 45.8)
**Alcohol use, %**		
Active	3.9	0.4
None	92.8	94.7
Weaned	3.2	4.9
**Drug addiction, %**		
Active	1.2	0.8
None	96.8	98.8
Weaned	1.9	0.4
Active opioid treatment	8.9	6.1
Active depression	13.6	8.2
**Previous surgery, n (%)**		
Hip	97 (23.9)	60 (24.6)
Spine	37 (9.1)	21 (8.6)
**Surgical approach, n (%)**		
Anterior	265 (65.3)	134 (53.6)
Posterolateral	141 (34.7)	116 (46.4)
**Femoral component, n (%)**		
Accolade	104 (25.6)	214 (85.6)
Anato	287 (70.7)	24 (9.6)
Others	15 (3.7)	12 (4.8)
**Liner, n (%)**		
MDM	50 (12.3)	54 (21.6)
PEX3	356 (87.7)	196 (78.4)

MDM, modular dual mobility.

### Radiological assessment

**Fig. 1:**
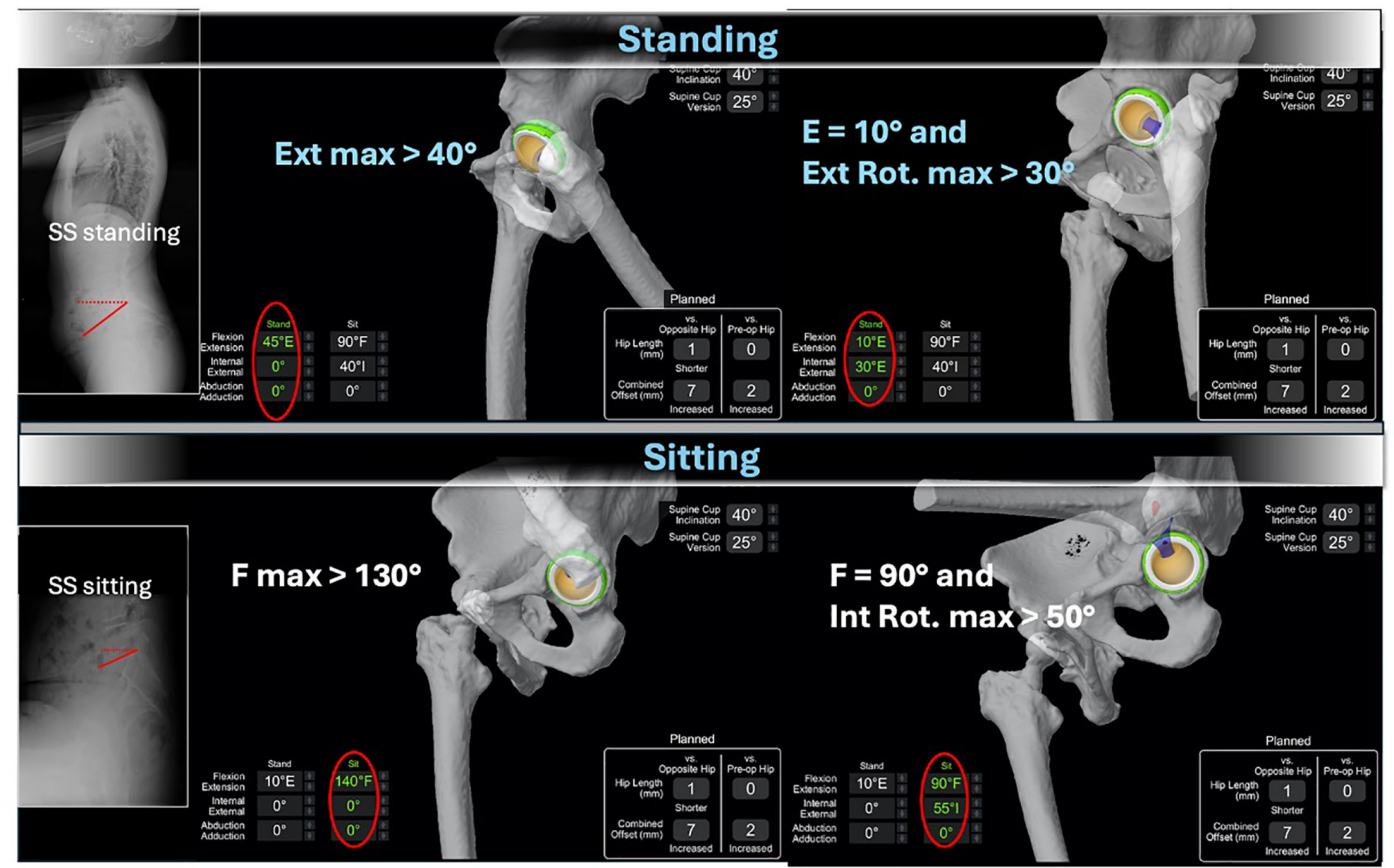
Collision detection model in the V4 software, integrating sacral slope measurements in both standing and sitting positions. The MAKO (Stryker, USA) CT-based planning enables optimization of implant positioning (acetabular component and femoral component) according to functional lumbopelvic kinematics. E, extension; F, flexion; Ext. Rot., external rotation; Int. Rot., internal rotation; SS, sacral slope.

Radiological assessment included a calibrated CT scan for MAKO 3D planning and standard pre- and postoperative radiographs (anteroposterior (AP) pelvic standing, AP and lateral hip, and full spine in AP and lateral views). In the V4 cohort, additional standing and seated lateral full-spine radiographs were obtained to measure sacral slope (SS), which was integrated into the collision model for 3D planning (Figure 1). Postoperative radiographs were used to assess outcomes.

### Surgical protocol

The cohort included three types of femoral components from Stryker: an uncemented anatomical design (Anato), an uncemented morphometric wedge-shaped design (Accolade II), and a cemented design (Exeter). Two acetabular component designs were used: Trident I (with or without Tritanium) and Trident II. Inserts were either highly cross-linked polyethylene (HXLPE) or dual-mobility (MDM). Bearing combinations included Biolox δ with X3 polyethylene for single- or dual-mobility cups, and metal-X3 polyethylene for dual-mobility constructs.

All procedures employed the MAKOplasty robotic system (Stryker). Preoperative CT-based 3D planning enabled adjustment of acetabular inclination (AI), acetabular anteversion (AA), centre of rotation (COR), global offset (GO), and intra-articular length (IAL) with 1° and 1 mm accuracy.^11^

### Surgical planning

With MAKO Total Hip 3.0 (V3), acetabular component positioning followed standard guidelines, aiming to avoid anterior overhang, preserve bone stock, and restore the centre of rotation using the contralateral hip as reference. Femoral component size was chosen for optimal fit and fill, with component version adjusted to a combined anteversion of 30° to 40° (typically 10° to 15°).^20^

Implant choice and head length were guided by restoration of IAL and GO, targeting ≤ 5 mm difference from the contralateral side.

**Fig. 2:**
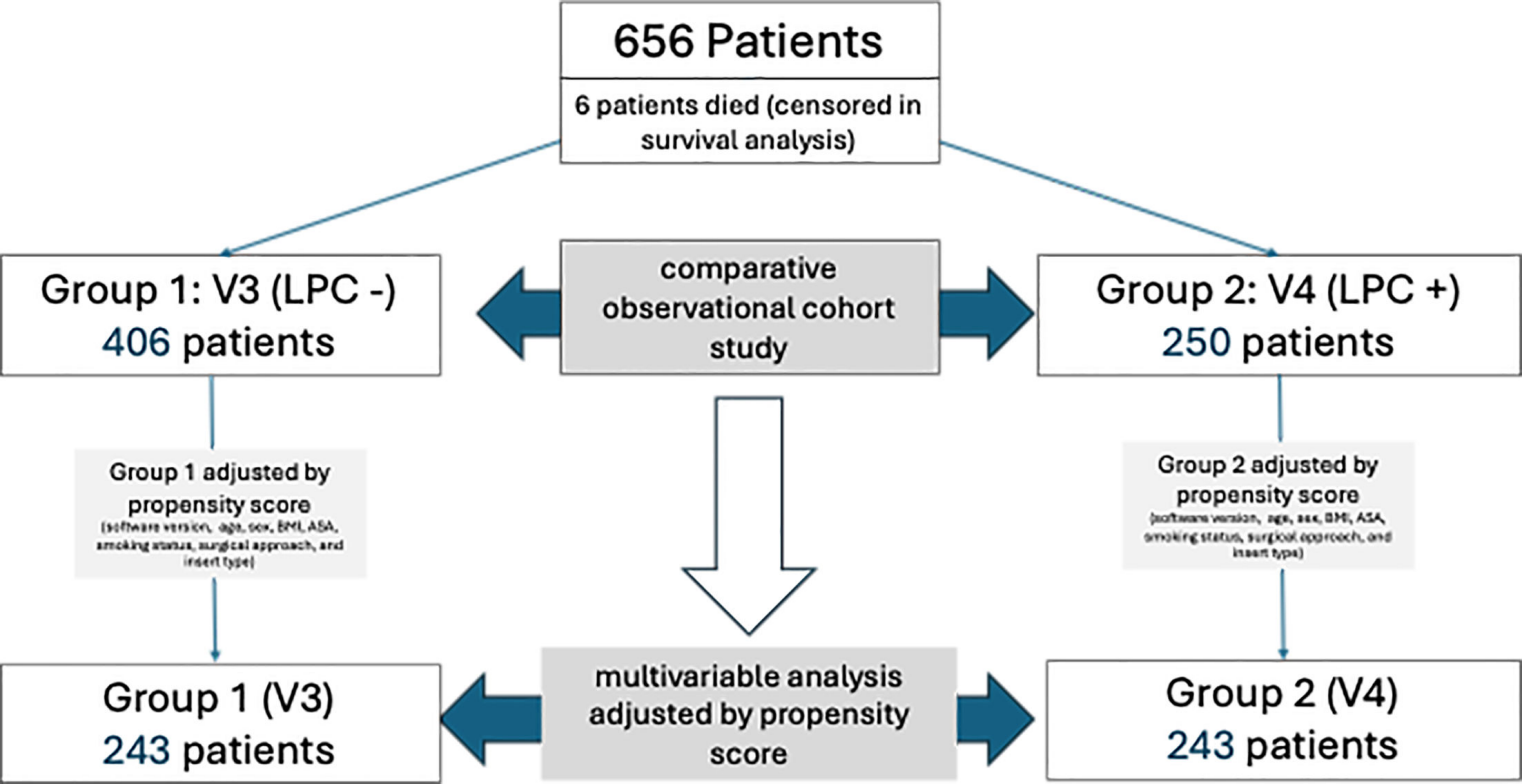
Study flowchart diagram of the 656 patients included in the cohort, with propensity-score matching yielding two comparable groups of 243 V3 and 243 V4 patients for multivariable analyses. LPC, lumbopelvic complex.

With MAKO Total Hip 4.0 (V4), the same principles applied, but standing and seated radiographs were used to incorporate pelvic tilt (sacral slope) into planning. Implant orientation, including femoral version, was refined through a collision model simulating functional extremes, with physiological reference thresholds drawn from the literature:^9,21^ standing with 40° hip extension and 0° rotation; standing with 10° extension and 30° external rotation; sitting with 130° flexion and 0° rotation; and sitting with 90° flexion and 50° internal rotation (Figure 1 and Figure 2). This model also guided implant selection (dual-mobility vs fixed bearing) and head length to minimize impingement and dislocation risk.

Patients treated before July 2022 underwent V3 planning, and those after this date underwent V4 planning.

### Surgical procedure

All procedures were performed with MAKO robotic assistance by two senior surgeons (PK, RC), using either the posterior Moore or the direct anterior Hueter approach (without a specialized table). In the V4 group, enhanced navigation of femoral component version was applied when the native version lay outside 10° to 15° or when the planned version differed by more than 3° (ΔNvF > 3°).

### Outcomes and complications assessment

Complications assessed included periprosthetic fractures, dislocations, periprosthetic joint infection, trochanteric fractures, and minor events (e.g. dysaesthesia, haematoma, heterotopic ossification, scar-related issues, femoral component migration < 2 mm, spinal decompensation, or medical complications). Reinterventions were recorded separately as revisions (implant exchange) or non-revision procedures. Complications were recorded and analyzed at the two-year follow-up, which was available for all included patients.

Functional outcomes were collected prospectively pre- and postoperatively using visual analogue scale (VAS) for pain (minimal clinically important difference (MCID) ≥ 2, patient acceptable symptom state (PASS) ≥ 4), Oxford Hip Score^22,23^ (OHS; PASS > 83/100), Forgotten Joint Score^24^ (FJS; PASS > 68/100), and Harris Hip Score^25^ (HHS; PASS ≥ 89/100).^26,27^

A comparative cohort analysis was performed between the V3 and V4 groups. A total of 656 patients were included; six patients died within the first postoperative year and were censored in the survival analyses. The study population consisted of 406 patients in the V3 cohort and 250 patients in the V4 cohort.

To minimize baseline differences between these two non-randomized cohorts, a propensity score was generated for each patient using a multivariable logistic regression model incorporating clinically relevant covariates (age, sex, BMI, ASA grade, smoking status, surgical approach, insert type, and software version). A 1:1 nearest-neighbour matching protocol without arthroplasty was applied, using a predefined caliper to optimize comparability between groups.

After matching, 243 patients from each cohort met balance requirements across all covariates and were included in the propensity score-adjusted comparative analysis. This matched population served as the basis for the multivariable modelling aimed at determining the independent effect of functional (V4) planning on postoperative complications and reoperation risk (study flowchart, Figure 2).

### Statistical analysis

Statistical analyses were performed using EasyMedStat (v. 3.36, EasyMedStat, France). Quantitative variables were described using means and medians, and qualitative variables using counts and proportions. Group comparisons were performed using Student’s t-test, Mann–Whitney U test, Kruskal–Wallis test, chi-squared test, or Fisher’s exact test, as appropriate. Survival was estimated using the Kaplan–Meier method and compared with the log-rank test. Correlations were assessed using Pearson or Spearman tests.

A multivariable logistic regression analysis was performed using ‘global complication’ (yes/no) as the dependent outcome. All clinically relevant variables (age, sex, BMI, ASA grade, smoking status, surgical approach, insert type, and software version) were included as predictors. Regression coefficients were estimated using the maximum likelihood method, and statistical significance was assessed using the Wald test.

Because the learning curves for both surgical approaches and implant adoption were completed prior to robotic implementation, and because the robotic learning curve is short and unlikely to influence outcomes, only variables that could realistically vary over time (approach distribution, femoral component type, insert type) were included in the propensity score and in the multivariable model to minimize temporal confounding.

The analysis was multivariable (multiple predictors, single outcome), not multivariate. Minor complications were grouped into a single composite endpoint and did not generate multiple independent outcomes. All patients had a minimum follow-up of two years, allowing the multivariable logistic regression to be performed on a complete two-year dataset without loss to follow-up.

Odds ratios (ORs) with 95% confidence intervals (CIs) were reported. Model assumptions were verified using the Belsley–Kuh–Welsch test for multicollinearity, the White test for heteroskedasticity, and the Shapiro–Wilk test for residual normality. A p-value < 0.05 was considered statistically significant.

## Results

The distribution of surgical approaches differed, with anterior approaches used in 265/406 of V3 cases (65.3%) and 134/250 of V4 cases (53.6%) (Table I). Implant use reflected temporal practice patterns: anatomical femoral components were more frequent in the V3 group (287/406; 70.7%), whereas wedge-shaped femoral components predominated in the V4 group (214/250; 85.6%). Modular dual-mobility liners were also more common in the V4 cohort (54/250; 21.6%).

**Fig. 3:**
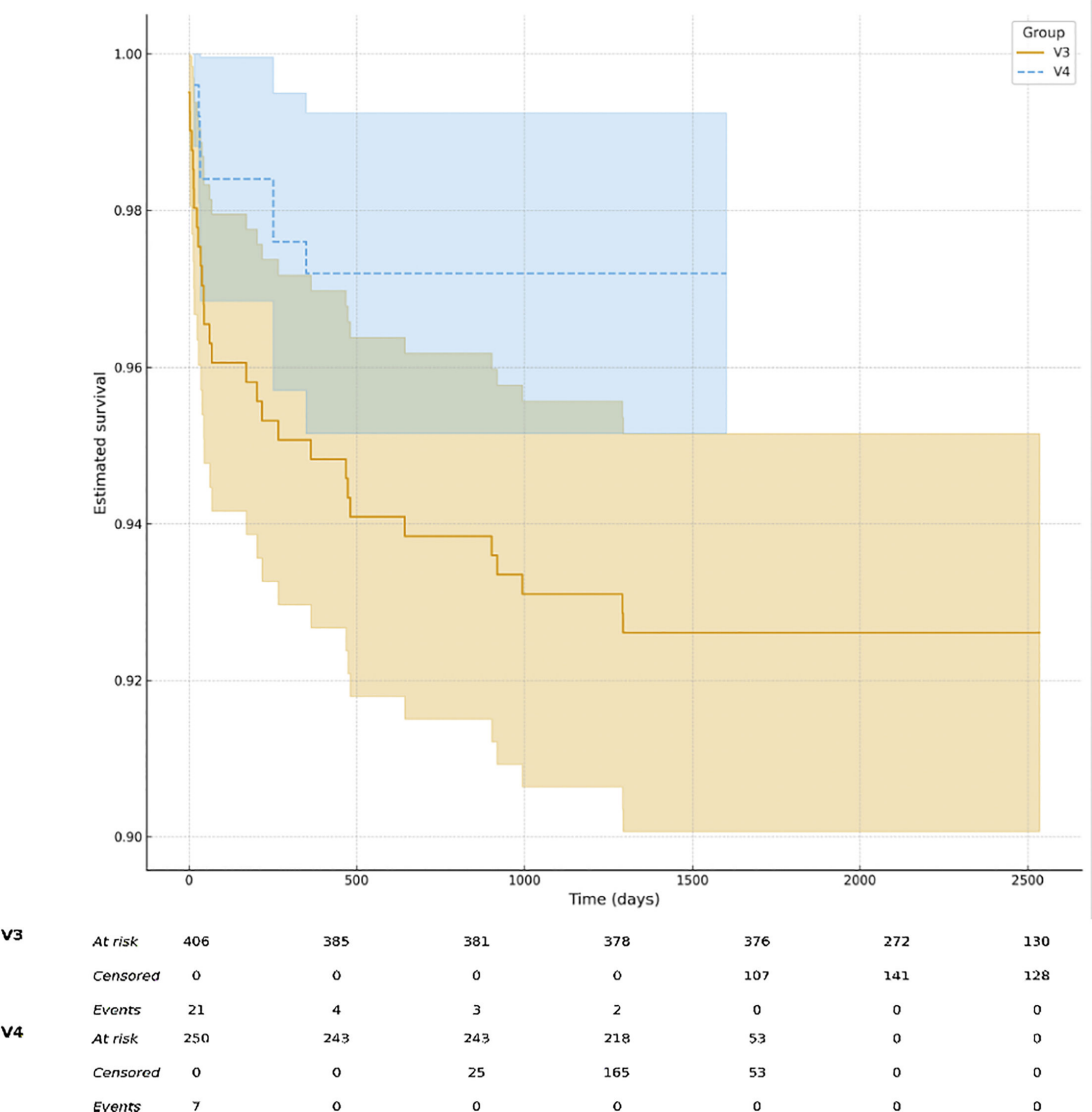
Kaplan-Meier curves showing dislocation-free survival after robotic-assisted total hip arthroplasty with V3 and V4 planning software. The V4 cohort demonstrated higher dislocation-free survival throughout follow-upcompared with the V3 cohort. Shaded areas indicate 95% confidence intervals.

**Fig. 4:**
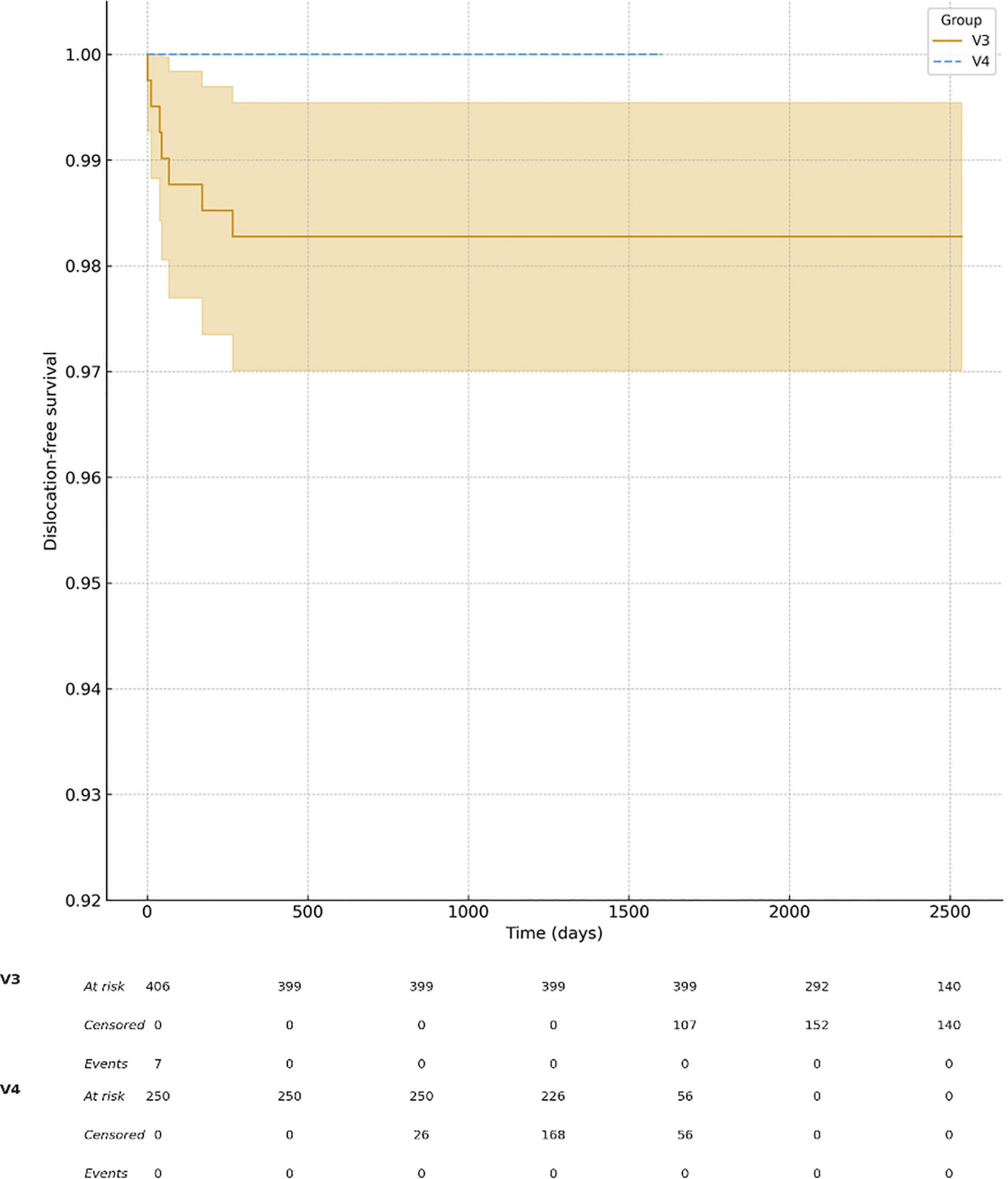
Kaplan-Meier curves showing reoperation-free survival after robotic-assisted THA with V3 and V4 planning software. The V4 cohort demonstrated no reoperations up to six years, while the V3 cohort showeda lower survival probability. Shaded areas indicate 95% confidence intervals.

**Fig. 5:**
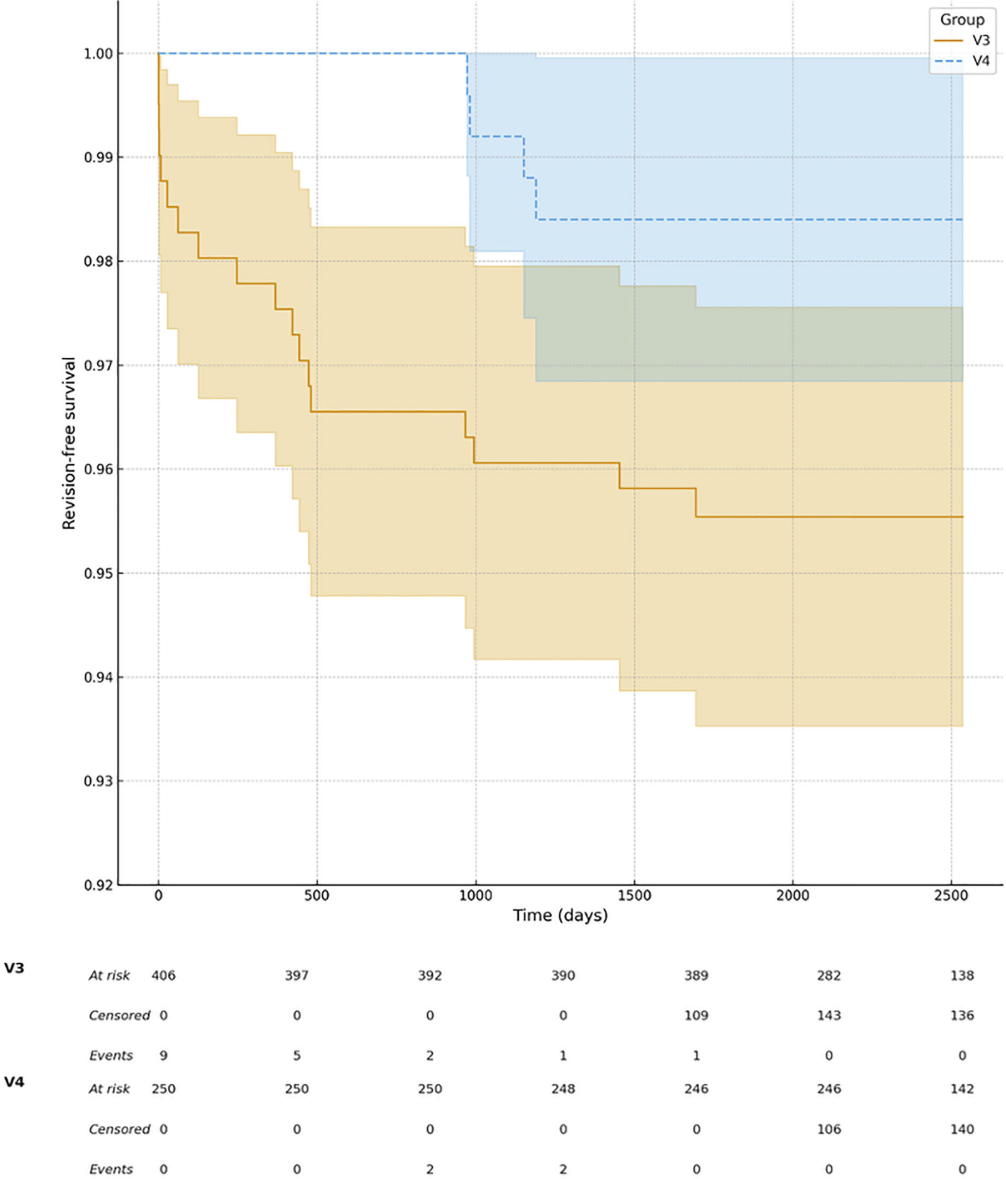
Kaplan-Meier analysis of revision-free survival after robotic-assisted total hip arthroplasty with V3 and V4 planning software. Shaded areas indicate 95% confidence intervals.

At two years, reoperation-free survival was higher in the V4 group (243/250; 97.2%) compared with V3 (382/406; 94.1%), as illustrated in Figures 3 to 5. Dislocation-free survival was 100% in the V4 cohort (250/250) and 98.3% in the V3 cohort (399/406). Revision-free survival was comparable between groups (V4, 246/250; 98.4% vs V3, 392/406; 96.5%).

Overall complications were reported in 7/250 V4 cases (2.8%) and 34/406 V3 cases (8.4%) (Table II). Reoperations occurred in 5/250 patients (2.0%)in the V4 cohort and 28/406 (6.9%) in the V3 cohort. Minor complications were also less frequent in V4. Notably, all dislocations (7/406, 1.7%) and all periprosthetic fractures (9/406, 2.2%) occurred in the V3 group. In the V3 group, seven dislocations occurred: two after an anterior approach and five after a posterolateral approach. Most had identifiable causes, including extensive spinal fusion (n = 4), gluteus maximus rupture (n = 1), post-traumatic sequelae (n = 1), and an osteophytic cam (n = 1). Periprosthetic fractures occurred in six cases following the anterior approach and in three cases following the posterolateral approach. Six fractures involved an anatomical femoral component, whereas three occurred with the Accolade II femoral component.

**Table II.** Postoperative complications in V3 and V4 cohorts, including dislocation, reoperation, and implant revision at two-year follow-up.

Variable	V3 group (n = 406)	V4 group (n = 250)	p-value
Postoperative complication, n (%)	34 (8.4)	7 (2.8)	0.007
**Complication types, n (%)**			0.365
Atraumatic dislocation	6 (1.5)	0 (0.0)	
Traumatic dislocation	1 (0.2)	0 (0.0)	
Great trochanter fracture	5 (1.2)	0 (0.0)	
Periprosthetic fracture	4 (1.0)	0 (0.0)	
Acute prosthetic joint infection	5 (1.2)	4 (1.6)	
Chronic prosthetic joint infection	5 (1.2)	2 (0.8)	
Others	8 (1.9)	1 (0.4)	
Dislocation (all types)	7 (1.7)	0 (0.0)	0.048
Reoperation	28 (7.0)	5 (2.1)	0.013
Implant revision	17 (4.2)	4 (1.6)	0.072

**Fig. 6:**
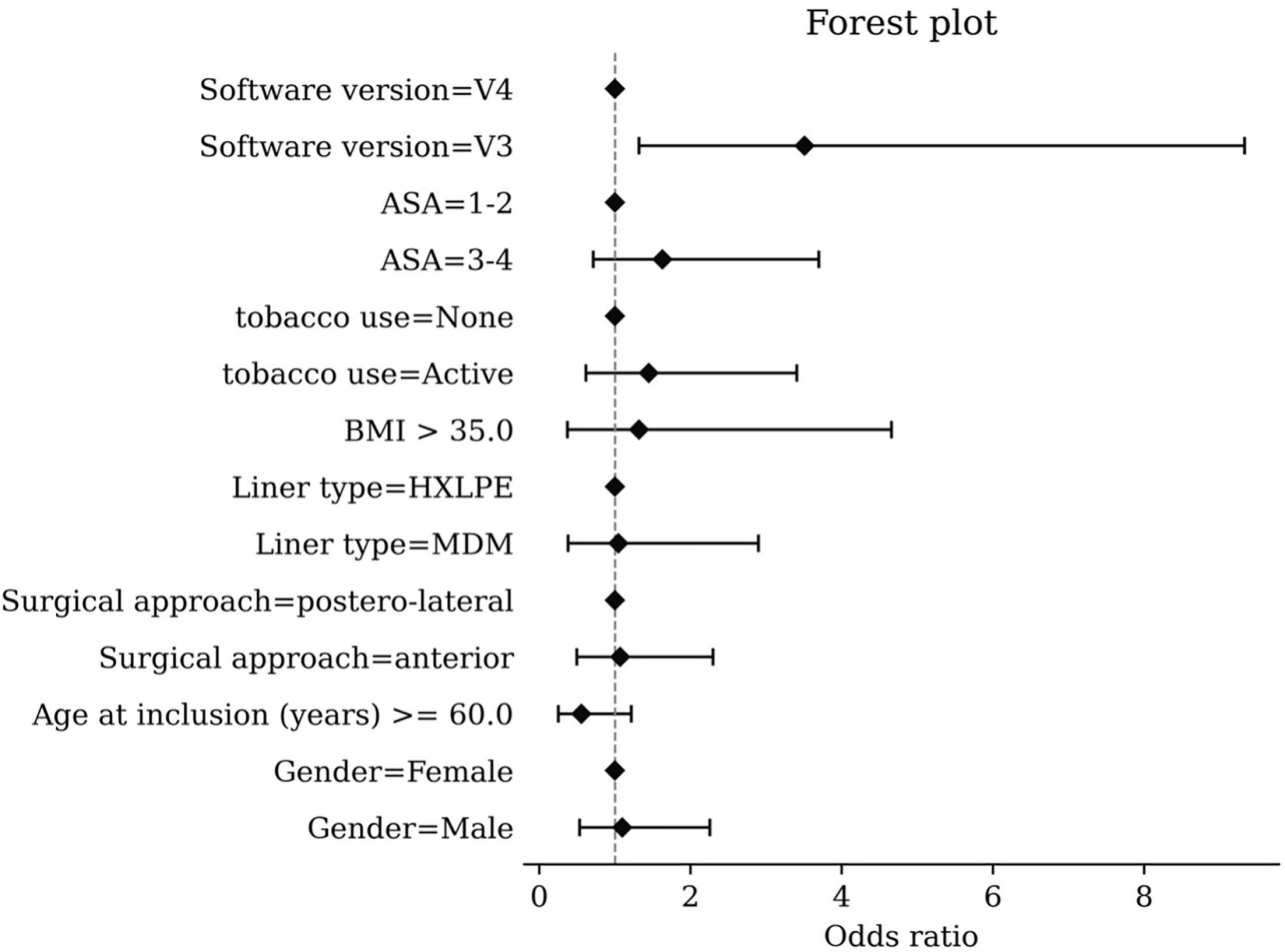
Forest plot of the multivariate logistic regression analysis assessing factors associated with revision. Variables included software version (V3 vs V4), American Society of Anesthesiologists (ASA) grade, tobacco use, BMI, liner type, surgical approach, age at inclusion, and sex. Odds ratios are shown with 95% CIs. HXLPE, highly cross-linked polyethylene. MDM, modular dual mobility.

Revision procedures were recorded in 4/250 patients (1.6%) in V4 and 17/406 (4.2%) in V3. In the multivariable analysis, the use of the V3 software was the only independent predictor of reoperation (OR 3.51, 95% CI 1.32 to 9.33). No significant associations were observed for age, sex, BMI, ASA grade, smoking status, surgical approach, or insert type (Table III and Figure 6).

**Table III.** Multivariate logistic regression including software version, ASA grade, tobacco use, BMI, liner type, surgical approach, age, and sex.

Variable	Reference category	Odds ratio (95% CI)	p-value*
Software version	V4	V3: 3.51 (1.32 to 9.33)	0.0119†
ASA grade	I to II	III to IV : 1.63 (0.72 to 3.70)	0.244
Tobacco use	None	Active: 1.45 (0.62 to 3.41)	0.392
BMI, kg/m^2^	≤ 35	> 35: 1.32 (0.37 to 4.66)	0.668
Liner type	HXLPE	MDM (dual-mobility): 1.05 (0.38 to 2.90)	0.922
Surgical approach	Posterolateral	Anterior: 1.07 (0.50 to 2.30)	0.857
Age at inclusion, yrs	≥ 60	< 60: 1.79 (0.82 to 3.93)	0.144
Sex	Female	Male: 1.10 (0.54 to 2.26)	0.799

* Wald test.

† Significant at p < 0.05.

ASA, American Society of Anesthesiologists; HXLPE, highly cross-linked polyethylene; MDM, modular dual mobility.

### Functional outcomes analysis

Postoperatively, HHS, OHS, and FJS were all statistically significantly higher in the V4 group (Table IV).

**Table IV.** Functional outcomes comparing V3 and V4 cohorts: hip and lumbar VAS, Harris Hip Score, Oxford Hip Score, and Forgotten Joint Score, with PASS thresholds.

Variable	V3 group (n = 406)	V4 group (n = 250)	p-value
Preoperative hip VAS	6.8 (1.7)	7.2 (1.6)	0.031
Preoperative lumbar VAS	5.0 ( 3.0)	4.4 (2.9)	0.019
Preoperative HHS	47.7 (15.1)	39.0 (14.0)	< 0.001
Preoperative OHS	39.0 (8.5)	39.7 (8.0)	0.408
Postoperative hip VAS	1.4 (2.1)	0.9 (1.6)	0.006
Postoperative lumbar VAS	2.4 (2.6)	2.5 (2.6)	0.658
Postoperative hip VAS ≥ PASS, %	77.3	87.7	0,009
Postoperative Harris Score ≥ PASS, %	44.2	61.2	< 0.001
Postoperative OHS	40.7 (7.7)	43.3 (5.7)	< 0.001
Postoperative OHS≥ PASS, %	70.1	86.5	< 0.001
Postoperative FJS	69.5 (28.5)	76.3 (25.2)	0.026
Postoperative FJS ≥ PASS, %	51.6	63.4	0.033

Data are shown as percentages or mean (SD).

FJS, Forgotten Joint Score; HHS, Harrris Hip Score; OHS, Oxford Hip Score; PASS, patient acceptable symptom state; VAS, visual analogue scale.

For the OHS, the mean improvement was 22.35 points (SD 9.16) in the V3 group and 24.3 points (SD 7.61) in the V4 group (p = 0.071). Although this difference approached statistical significance, it did not reach the minimally important difference (MID)^28^ of 5 points for the OHS, indicating that the between-group difference was not clinically meaningful.

PASS thresholds were also achieved more frequently in the V4 group for the HHS, OHS, and FJS. At 12 months, lumbar VAS did not differ (2.44 vs 2.51), whereas hip pain VAS was lower in V4 (0.88 vs 1.44), with more patients reaching PASS criteria (87% vs 77%).

## Discussion

This study demonstrated that incorporating lumbopelvic kinematics into preoperative 3D planning significantly reduced early complications after robot-assisted THA. Patients planned with V4 had fewer overall complications, no dislocations or periprosthetic fractures, and superior reoperation-free survival at two years. Multivariate analysis confirmed that software version was the only independent predictor of reoperation, with V3 associated with a 3.5-fold higher risk. Functional outcomes were also superior with V4, with higher rates of patients reaching PASS thresholds across all patient-reported outcome measures (PROMs).

Robotic assistance in THA has consistently improved the accuracy and reproducibility of implant positioning, particularly for cup inclination and anteversion, leg length, and offset restoration.^11,29,30^ However, conventional CT-based planning does not account for functional pelvic tilt or spinal stiffness, both of which directly affect cup orientation and stability. The integration of sacral slope in standing and sitting postures, as introduced with V4, enables a patient-specific collision model that better reflects real-life functional positions. Previous publications have demonstrated that pelvic inclination, assessed in both standing and sitting positions, plays a key role in hip kinematics and directly influences cup orientation. Our results confirm that such functional planning is protective against reoperation and instability, moving beyond static planning towards a personalized ‘functional safe zone’^8,18,27^

The V4 version of the planning software differs from V3 by incorporating patient-specific functional information. While V3 relies exclusively on supine CT-based measurements to define implant orientation, V4 additionally integrates pelvic tilt values in both standing and seated positions, allowing the software to simulate changes in acetabular orientation across postures. V4 also provides collision detection based on hip range of motion to identify impingement risk and estimate functional combined anteversion. These additional inputs and outputs enable a functional, rather than purely static, definition of cup positioning. Similar concepts of posture-dependent planning and collision modelling have been described in the literature and are increasingly recognized as relevant for reducing instability risk.^10,31^

The classical concept of the ‘safe zone’,^32^ although widely adopted, has shown important limitations: dislocations may occur within the defined zone, while stable hips may be found outside it. These inconsistencies prompted progressive refinements of angular thresholds and led to the emergence of the ‘functional safe zone’, which incorporates lumbopelvic mobility in both standing and sitting positions. Tezuka et al^33^ introduced the Combined Sagittal Index (CSI) as a tool to identify patients at risk of prosthetic impingement even when conventional positioning criteria are satisfied. More recently, O’Connor et al^10^ proposed the concept of functional combined version, integrating postural acetabular orientation with femoral version. In their series of 100 robotic-assisted THAs, a combined version between 35° and 55° optimized range of motion and minimized impingement, particularly in patients with spinal stiffness.

Together, these findings illustrate a paradigm shift from static angular targets to functional, patient-specific criteria. Our study adds evidence that robotic assistance, when combined with functional planning, provides this level of precision by tailoring implant orientation to individual lumbopelvic kinematics, which may explain the superior outcomes observed in the V4 cohort.

The overall dislocation rate in our series was higher than that reported in most robotic-assisted THA studies, which typically show a reduction in instability. Shaw et al^34^ reported a dislocation rate of 0.6%, while Bendich et al^35^ found a significantly reduced revision risk for dislocation compared with conventional techniques (OR 0.3). Conversely, Swartz et al^36^ reported a two-year dislocation rate of 1.66%, which aligns closely with our findings. This discrepancy may reflect our case-mix, which included many patients with spinal fusions or pre-existing spinal pathology. In fact, all dislocations occurred in the V3 group and primarily in such high-risk patients, underscoring both the vulnerability of this population and the protective effect of functional planning with V4, in which no dislocations were recorded.

Previous studies have highlighted the impact of patient- and surgery-related factors such as obesity, diabetes, smoking, spinal stiffness, and implant design on revision risk.^4^ In our cohort, none of these variables were independently associated, likely reflecting the predominant effect of functional planning. Instead, the only independent predictor identified was the type of preoperative planning: functional planning (V4), which integrates lumbopelvic kinematics, appeared as a protective factor against reoperation. This supports recent literature on the ‘functional safe zone’, which incorporates postural pelvic tilt and combined version to reduce instability and mechanical complications.^10,31,33^

Regarding PROMs, prior studies have shown improved short- and mid-term outcomes when implant positioning optimizes the centre of rotation, offset, and intra-articular length.^11,13^ However, no previous work has specifically examined postoperative complications at one year when comparing two planning strategies, one incorporating pelvic tilt and one not. Earlier comparisons of robotic-assisted with conventional THA produced inconsistent PROM results^37^ likely because they relied on static planning alone. Our findings therefore suggest that robotic assistance improves outcomes only when combined with functional planning. Persistent lumbar pain in our series indicates that more specific assessment tools, such as the Oswestry Disability Index, would be needed to evaluate changes in spinal function over time.

This study has several limitations. Despite prospective data collection, the retrospective analysis classifies it as a Level IV cohort, with inherent risks of bias. Follow-up was limited to two years, which remains insufficient to assess long-term implant survival or late complications. Outcomes in the V4 group may partly reflect both technological advances and the surgical team’s learning curve, as well as differences in implant selection, particularly the increased use of dual-mobility cups, which may independently reduce the risk of dislocation. Nevertheless, these cups were selected according to collision-model simulations, reinforcing the relevance of functional planning.

Although femoral component design may influence combined anteversion, the marked imbalance in femoral component types between groups prevented any meaningful comparison of stem-related complications; however, femoral component anteversion was consistently controlled to remain within Dorr’s recommended range.^20^

Although this study follows a natural-experiment design, the learning curves for both surgical approaches and implant adoption had already been completed before the introduction of robotic-assisted THA. The robotic learning curve itself is known to be short in the literature and is unlikely to have influenced the results. The only factors that may have evolved during the V3 to V4 transition are the refinement of preoperative planning and increased surgeon familiarity with functional lumbopelvic parameters, which could represent a residual bias despite statistical adjustment.

Finally, the study may be underpowered for detecting differences in rare adverse events, particularly dislocations and early failures. Even though the overall cohort is relatively large, the low event rate limits the statistical power of sub-group analyses and may mask small but clinically meaningful differences between groups.

Strengths include a large consecutive cohort with prospectively collected data, no patient exclusions, and robust multivariable modelling. This enhances internal validity and external generalizability, supporting the relevance of our findings to routine practice.

In conclusion, integrating pelvic tilt and the functional variations of the lumbopelvic complex into 3D robotic planning significantly reduces early complications and improves short-term outcomes in THA. In our multivariate analysis, functional planning (V4) incorporating lumbopelvic kinematics was the only independent protective factor against reoperation, underscoring the clinical importance of moving beyond static CT-based methods. By tailoring implant orientation to each patient’s functional anatomy, this approach defines a safer, more personalized ‘functional safe zone’, maximizing stability and mobility while minimizing impingement and dislocation.

**Take home message**

- This study demonstrates that incorporating lumbopelvic kinematics into robotic-assisted total hip arthroplasty (THA) significantly reduces early complications, including dislocation and reoperation.

- Functional 3D planning enables a more patient-specific implant positioning strategy, improving stability and short-term outcomes. These findings support a shift from static to functional planning to optimize results in contemporary THA.

## Data Availability

The datasets generated and analyzed in the current study are not publicly available due to data protection regulations. Access to data is limited to the researchers who have obtained permission for data processing. Further inquiries can be made to the corresponding author.
